# The association between visual function and retinal structure in chronic central serous chorioretinopathy

**DOI:** 10.1038/s41598-017-16339-9

**Published:** 2017-11-24

**Authors:** Aya Sugiura, Ryosuke Fujino, Nobuko Takemiya, Kimiko Shimizu, Masato Matsuura, Hiroshi Murata, Tatsuya Inoue, Ryo Obata, Ryo Asaoka

**Affiliations:** Department of Ophthalmology, University of Tokyo Graduate School of Medicine, Tokyo, Japan

## Abstract

The purpose of the current study was to investigate the association between visual function and retinal structure in central serous chorioretinopathy (CSC). In 22 eyes of 22 chronic CSC patients with serous retinal detachment at the macula, retinal sensitivity was measured using MP3 microperimetry (NIDEK, Japan) and mean sensitivity within two degrees (MS_2_), four degrees (MS_4_) and six degrees (MS_6_), as well as foveal sensitivity (MS_0_), were calculated. Retinal structure was measured using optical coherence tomography (OCT, Spectralis, Heidelberg). The relationship between visual function (LogMAR best-corrected visual acuity [LogMAR VA] and MS_0_, MS_2_, MS_4_, MS_6_) and serous retinal detachment height at the fovea (SRDH), central retinal thickness (CRT), macular volume (MV) and central choroidal thickness (CCT) was investigated. There were significant negative correlations between LogMAR VA and MS_0_ (p = 0.016), MS_2_ (p = 0.049). No significant relationship was observed between LogMAR VA and SRDH (p = 0.97) although there was a significant negative correlation between SRDH and MS_2_ (p = 0.028), MS_4_ (p = 0.049), MS_6_ (p = 0.023). In conclusion, in CSC, serous retinal detachment was significantly correlated with retinal sensitivity measured with MP3, but not with LogMAR VA.

## Introduction

Central Serous Chorioretinopathy (CSC) is characterized by serous retinal detachment (SRD) through a defect of the retinal pigmented epithelium (RPE) layer^1^. The detailed physiopathologic mechanism of the disease remains unclear^2–6^, but leakage of vascular fluid through a focal leak in the RPE into the subretinal space as a consequence of hyperpermeability in the underlying choroid may be the principal cause; the disorder is commonly related to an exposure to systemic corticoids^7–10^.

Functional evaluation of CSC is usually carried out using visual acuity (VA). Most initial retinal detachments in CSC resolve spontaneously^11,12^; however recurrence of CSC is common and patients with chronic CSC often describe visual symptoms, such as decreased retinal sensitivity, metamorphopsia, and central scotoma^13–15^, with the reappearance of SRD, even if VA is preserved.

Microperimetry, also known as fundus related perimetry, is a type of visual field (VF) test that enables the detailed assessment of macular retinal sensitivity. Previous studies have highlighted the usefulness of this type of perimetry^16–27^, but all these studies used microperimetry to evaluate the effects of treatment interventions on macular retinal sensitivity in a relatively short period after the treatment, and to date, no study has revealed the usefulness of retinal sensitivity with microperimetry in chronic CSC. Microperimetry is useful to assess retinal sensitivity at an exact location, because it is possible to choose test locations from a corresponding fundus image. One issue, however, is eye movements during the test. In glaucoma there is clear evidence that eye movements affect the accurate perimetric evaluation of retinal sensitivity^28–30^. The MP-3 microperimeter (Nidek Co.ltd., Aichi, Japan) is equipped with an auto-tracking system that enables fine adjustments of the test location depending on the eye position during the VF measurement. Thus, it is expected that a more accurate measurement of retinal sensitivity is achieved with the MP-3. It is of interest to investigate the degree of correlation between structural change and VA or MP-3 sensitivity measurements in chronic CSC patients.

In the current study, retinal sensitivity was measured with MP-3 in patients with chronic CSC. Structural measurements of patients’ retinas were also made using optical coherence tomography (OCT). Then the structure-function relationship was investigated for MP-3 and OCT measurements, and compared against the relationship between VA and OCT measurements.

## Method

The study was approved by the Research Ethics Committee of the Graduate School of Medicine and Faculty of Medicine at The University of Tokyo. Written informed consent was given by patients for their information to be stored in the hospital database and used for research. This study was performed according to the tenets of the Declaration of Helsinki.

### Subjects

Twenty two eyes (11 right and 11 left eyes) of 22 chronic CSC patients (16 males and 6 females) were included in the study. Chronic CSC was defined as those with CSC for at least three months. Patients were diagnosed based on OCT, fluorescein angiography (FA) and Indocyanine green angiography (ICGA). All patients were prospectively recruited at the retina clinic in The University of Tokyo Hospital. Each patient underwent VF testing with MP-3 and OCT, carried out on the same day.

All patients enrolled in the study fulfilled the following criteria: (1) CSC was the only disease causing VF damage; (2) chronic CSC defined as SRD that had not resolved itself within 3 months; (3) measured refraction was between −6 and + 6 diopter (D).

### MP-3 measurement

**Figure 1:**
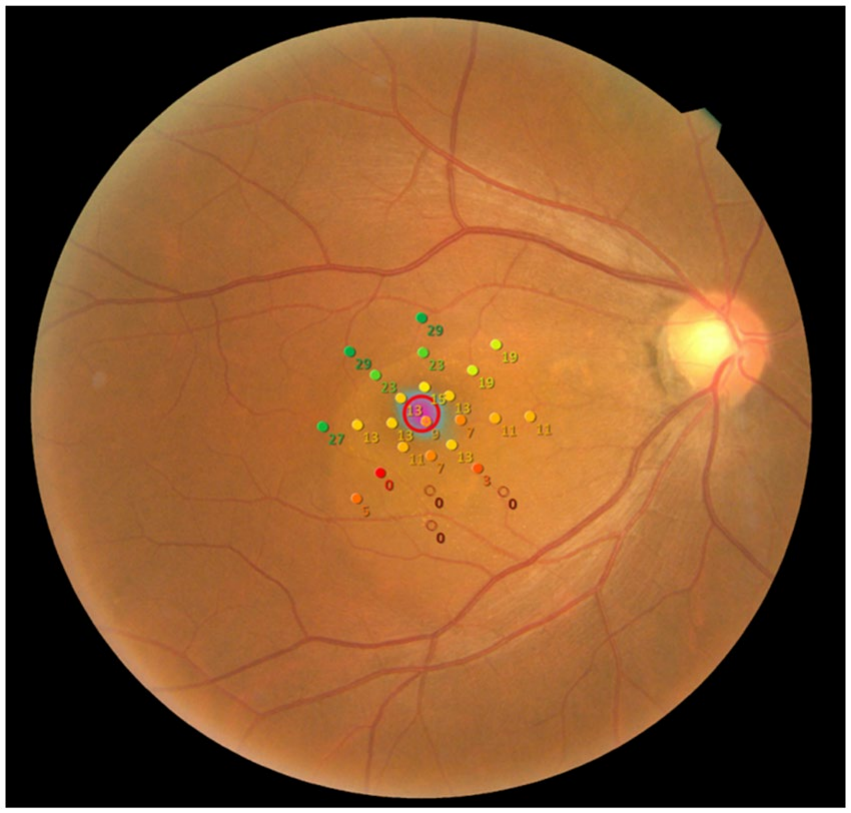
Example VF measurement with the MP-3 Perimetry results of a 53 years old male with CSC. VF: visual field, CSC: Central Serous Chorioretinopathy.

All patients had a pupil size that was larger than 4 mm in diameter, which is required for the MP-3 measurement. Similar to the Humphrey Field Analyzer (HFA) VF measurement, the MP-3 test is based on a 4–2 full threshold staircase strategy using a Goldmann III stimulus size. The 25 measured test points in the MP-3 are shown in Fig. 1. In the MP-3 test, the fixation target is a 1° diameter red circle, and the background luminance is set at 31.4 asb. The maximum luminance of the MP-3 is 10,000 asb, and the stimulus dynamic range is between 0 and 34 dB. In this study, MP-3 examinations were performed in a dimly lit room. Only reliable VFs were used in analyses, defined as a fixation loss (FL) rate < 20% and a false-positive (FP) rate < 15%. Using the obtained retinal sensitivities, the mean sensitivity at the fovea (MS_0_), within two degrees (MS_2_), four degrees (MS_4_), and six degrees (MS_6_) were calculated.

### OCT measurement

Spectral domain (SD) OCT data were obtained using the Spectralis OCT (Heidelberg Engineering, Heidelberg, Germany). All OCT images consisted of line scans (horizontal and vertical B-scans), raster scans, and topographic mapping. Line scans were created by averaging up to 100 B-scans (768 A-scans per B-scan) within 30°. The raster scan was performed using 25 B-scans (768 A-scans per B-scan) in a 30° × 20° area. The line scans, raster scans, and topographic mapping were performed on each eye.

**Figure 2:**
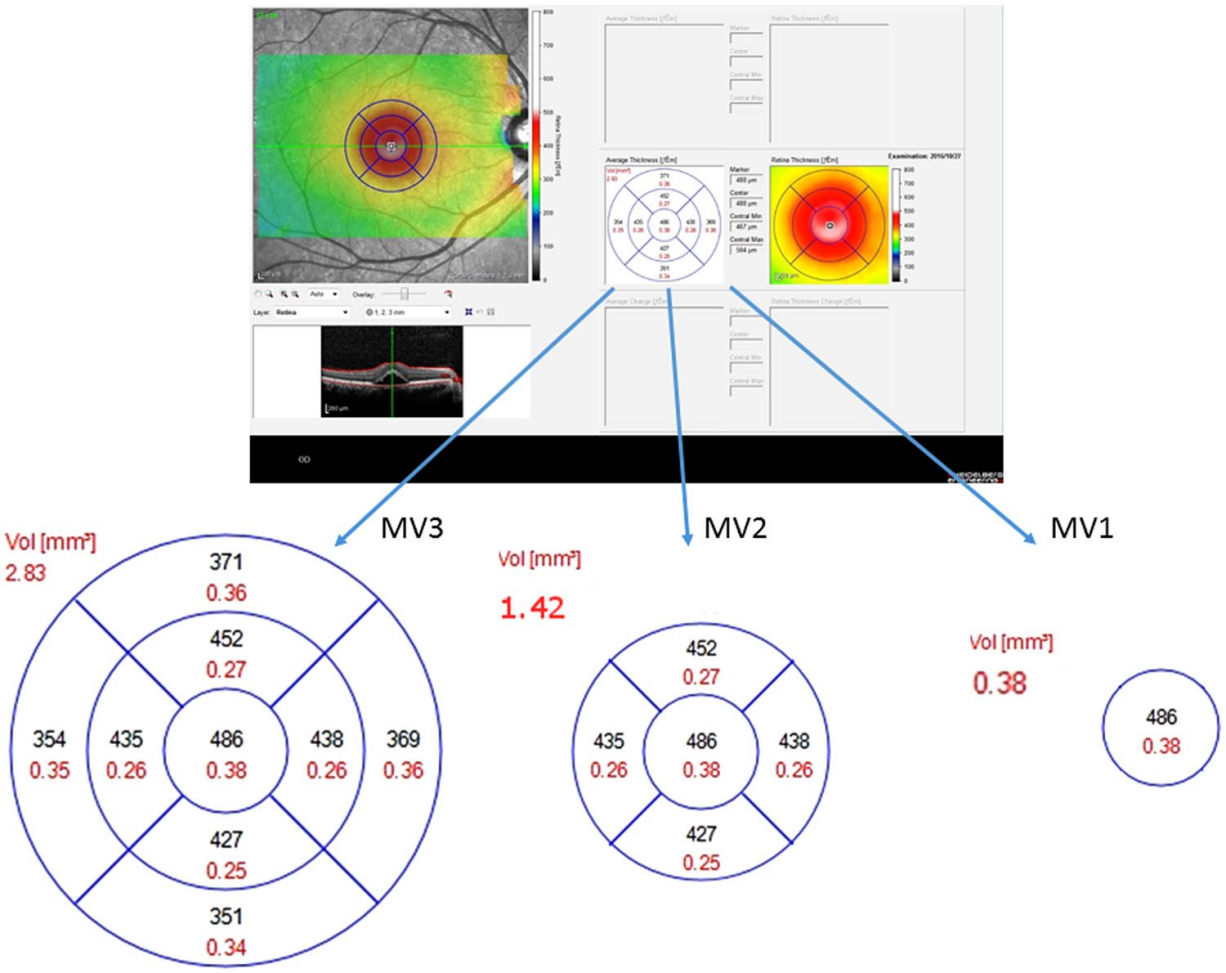
Macular volume measurement Macular volume within 1 mm diameter (MV_1_), 2 mm diameter (MV_2_), 3 mm diameter (MV_3_) were evaluated using OCT measurement OCT: optical coherence tomography.

Central retinal thickness (CRT), macular volume within a 1 mm diameter (MV_1_), 2 mm diameter (MV_2_) and 3 mm diameter (MV_3_), serous retinal detachment height (SRDH) and central choroidal thickness (CCT) were measured using the enhanced depth imaging mode (see Fig. 2). MV_1_, MV_2_, and MV_3_ were calculated based on the thickness between the inner limiting membrane and Bruch’s membrane. SRDH was measured as the distance from the outer surface of the neurosensory retina to the inner surface of the RPE, at fovea. MV_1_, MV _2_, and MV_3_ were inferred using the Spectralis OCT’s built-in function (Fig. 1). SRDH and CRT were estimated by two examiners (AS and RF) and the coefficient of variation and the intraclass correlation coefficient were calculated. The average SRDH value was used in all analyses. Superimposing 1, 2, and 3 mm diameter grid obtained from Spectralis OCT on MP-3 fundus image^31^, MS_0_ (mean sensitivity at 0 degree) and MS_2_ (mean sensitivity not outer than 2 degrees) correspond to the region of MV_1_, MS_4_ (mean sensitivity not outer than 4 degrees) corresponds to the MV_2_ area, and MS_6_ (mean sensitivity not outer than 6 degrees) corresponds to the MV_3_ area.

### Statistical analyses

The relationship between LogMAR VA and MP-3 sensitivity was analyzed using linear regression. The relationship between SRDH, CRT, CCT, MV_1_, MV_2_, LogMAR VA, MS_0_, MS_2_, and MS_4_ were also analyzed using the linear regression. Also, a number of other relationships were investigated between: (1) LogMAR VA, and the values of MV_1_ and SRDH; (2) MS_0_ and MS_2_, and the values of MV_1_, and SRDH; (3) MS_4_, and the values of MV_2_, SRDH; (4) MS_6_, and the values of MV_3_ and SRDH.

All analyses were performed using the statistical programming language ‘R’ (R version 3.1.3; The Foundation for Statistical Computing, Vienna, Austria).

## Results

Characteristics of the study subjects are summarized in Table 1. MS_0_, MS_2_, MS_4_, and MS_6_ were 16.2 ± 5.2 [4.0 to 25.0] (mean ± standard deviation: SD) [range], 19.6 ± 5.7 [4.9 to 28.8], 20.8 ± 6.0 [4.9 to 29.0], 21.7 ± 5.2 [11.4 to 29.1], respectively. The disease duration was 17.4 ± 9.8 [3 to 33] months; this data was not collected in two eyes and calculated only from 20 eyes. The SRF did not show a great deal of change throughout the follow up period. The coefficient of variation for CRT between the two observers was 1.1 ± 1.8 [0.0 to 6.3] μm. The intraclass correlation coefficient was 99.7% (95% confidence interval: 99.2 and 99.9%). Amongst the 22 eyes studied, 4 eyes had been treated with photodynamic therapy, 8 eyes had been treated with laser photocoagulation and none of the eyes had both treatments prior to this study. None of the patients had received laser treatment within 3 mm of the macula.

**Table 1:** Subjects demographics.

variables	value
age (years), mean ± SD [range]	54.6 ± 12.3 [37 to 77]
gender, male:female	16:6
eye, righ:left	11:11
LogMAR VA, mean ± SD [range]	0.17 ± 0.25 [−0.079 to 0.70]
MS_0_ (dB), mean ± SD [range]	16.2 ± 5.2 [4.0 to 25.0]
MS_2_ (dB), mean ± SD [range]	19.6 ± 5.7 [4.9 to 28.8]
MS_4_ (dB), mean ± SD [range]	20.8 ± 6.0 [4.9 to 29.0]
MS_6_ (dB), mean ± SD [range]	21.7 ± 5.2 [11.4 to 29.1]
CRT (μm), mean ± SD [range]	331.1 ± 93.1 [213.5 to 507.5]
SRD (μm), mean ± SD [range]	132.6 ± 94.2 [10.0 to 324.0]
MV_1_ (μm^3^), mean ± SD [range]	0.27 ± 0.070 [0.18 to 0.41]
MV_2_ (μm^3^), mean ± SD [range]	1.16 ± 0.24 [0.82 to 1.71]
MV_3_ (μm^3^), mean ± SD [range]	2.58 ± 0.45 [1.92 to 3.69]
CCT (μm), mean ± SD [range]	317.8 ± 79.4 [185.0 to 436.0]
Disease duration (months), mean ± SD [range]	17.4 ± 9.8 [3 to 33]

SD: standard deviation, VA: visual acuity, MS_0_: mean sensitivity at fovea, MS_2_: mean sensitivity within two degrees, MS_4_: mean sensitivity within four degrees, MS_6_: mean sensitivity within six degrees, SRDH: serous retinal detachment height, CRT: central retinal thickness, MV_1_: macular volume within 1 mm diameter, MV_2_: macular volume within 2 mm diameter, MV_3_: macular volume within 3 mm diameter, CCT: central choroidal thickness, Disease duration was collected from 46 eyes of 40 patients.

There was a significant relationship between LogMAR VA and MS_0_ (coefficient was −0.024, p = 0.016) and MS_2_ (−0.019 and 0.049), but not with MS_4_ (−0.014 and 0.12) and MS_6_ (−0.013 and 0.21). SRDH was significantly related to: CRT (coefficient = 0.94, p < 0.0001, linear regression, MV_1_ (coefficient = 803.5 and p = 0.0035), MV_2_ (coefficient = 246.4 and p = 0.0014) and MV_3_ (coefficient = 125.0 and p = 0.0032).

**Figure 3:**
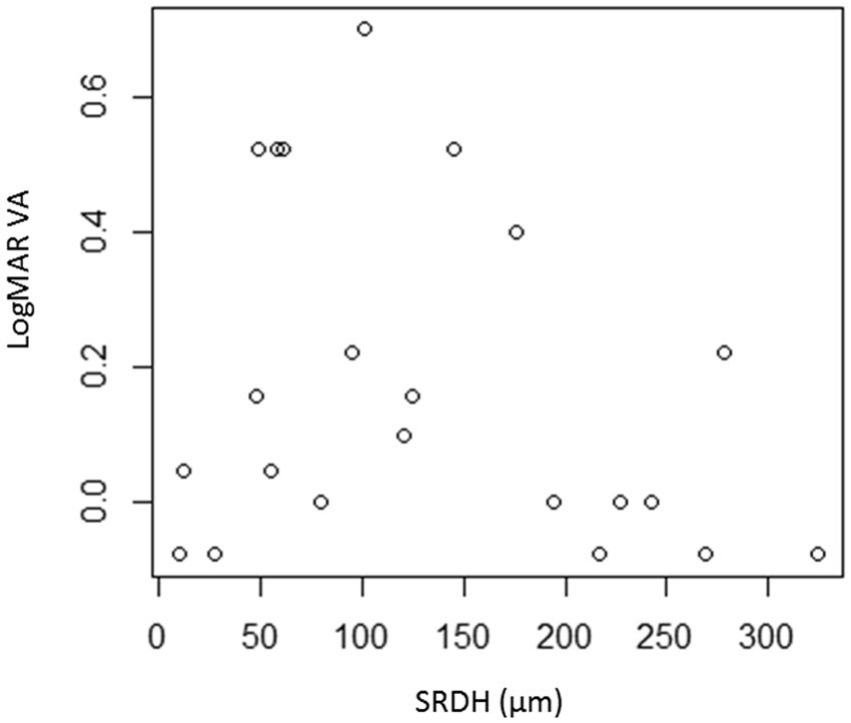
The relationship between LogMAR VA and SRDH No significant relationship was observed between LogMAR VA and SRDH (coefficient = −7.1 × 10^−4^, p = 0.22, linear regression). SRDH: serous retinal detachment height.

**Figure 4:**
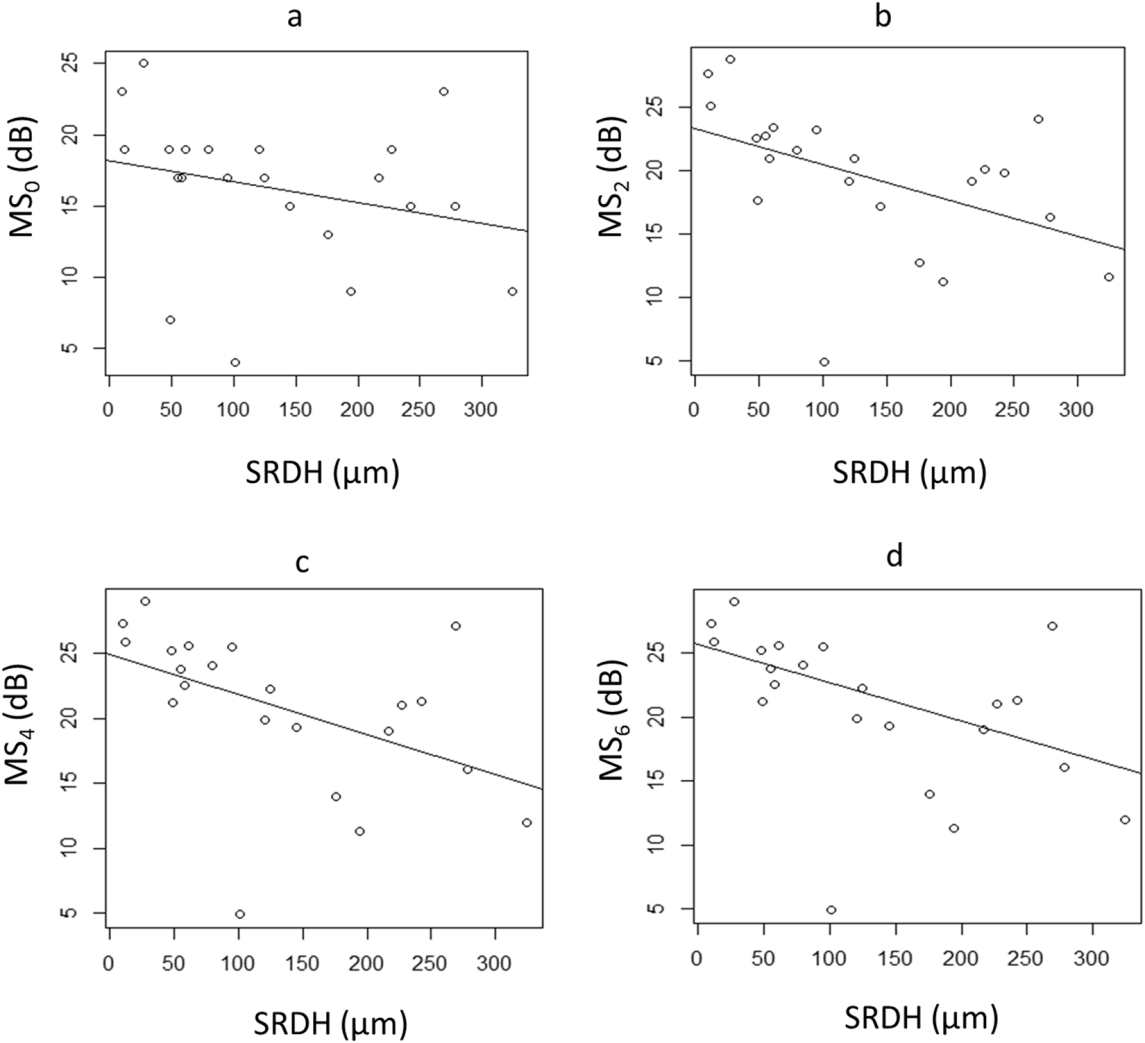
The relationship between MP-3 measured retinal sensitivities and SRDH A: MS_0_ and SRDH, significant relationship was not observed (coefficient = −0.015, p = 0.24), B: MS_2_ and SRDH, significant relationship was observed (coefficient = −0.029, p = 0.027), C: MS_4_ and SRDH, significant relationship was observed (coefficient = −0.031, p = 0.024), and D: MS_6_ and SRDH, significant relationship was observed (coefficient = −0.030, p = 0.0087). SRDH: serous retinal detachment height, MS_0_: mean sensitivity at fovea, MS_2_: mean sensitivity within two degrees, MS_4_: mean sensitivity within four degrees, MS_6_: mean sensitivity within six degrees.

Figure 3 and Fig. 4 show the scatterplots between SRDH and LogMAR VA, MS_0_, MS_2_, MS_4_, and MS_6_. In these univariate analyses, MS_2_, MS_4_, and MS_6_ were significantly related to SRDH (p < 0.05, linear regression). LogMAR VA was not significantly correlated to SRDH (coefficient = −7.1 × 10^–4^, p = 0.22). MS_0_ was not significantly correlated to SRDH (coefficient = −0.015, p = 0.24).

Table 2 shows the results of multivariate analyses. LogMAR VA was not significantly related to SRDH (p = 0.97). MS_0_ was also not significantly related to SRDH (p = 0.11). On the other hand, MS_2_ (coefficient = −0.035, p = 0.028), MS_4_ (coefficient = −0.035, p = 0.017) and MS_6_ (coefficient = −0.033, p = 0.023) were significantly correlated with SRDH. Because SRDH and CRT was very closely corelated, (R = 0.94, p < 0.001), CRT was not included in these analyses.

**Table 2:** Relationship between LogMAR VA and microperimetry measured retinal sensitivities and, OCT-measured thicknesses in the retina.

	LogMAR VA			MS_0_			MS_2_		
	coefficient	SE	p value	coefficient	SE	p value	coefficient	SE	p value
SRDH	2.6 × 10^−5^	6.7 × 10^−4^	0.97	−0.025	0.015	0.11	−0.035	0.015	0.028
MV_1_	−1.7	0.90	0.078	22.9	19.9	0.27	16.1	20.1	0.43
MV_2_	—	—	—	—	—	—	—	—	—
MV_3_	—	—	—	—	—	—	—	—	—
	**MS**_**4**_			MS_6_					
	**coefficient**	**SE**	**p value**	**coefficient**	**SE**	**p value**			
SRDH	−0.035	0.017	0.049	−0.033	0.013	0.023			
MV_1_	—	—	—	—	—	—			
MV_2_	2.60	6.43	0.69	—	—	—			
MV_3_	—	—	—	0.93	2.74	0.74			

VA: visual acuity, OCT: optical coherence tomography, SE: standard error, MS_0_: mean sensitivity at fovea, MS_2_: mean sensitivity within two degrees, MS_4_: mean sensitivity within four degrees, MS_6_: mean sensitivity within six degrees, SRDH: serous retinal detachment height, MV_1_: macular volume within 1 mm diameter, MV_2_: macular volume within 2 mm diameter, MV_3_: macular volume within 3 mm diameter.

CCT was not significantly related to these measurements: CRT (coefficient = 0.49, p = 0.056), MV_1_ (coefficient = 3.4 × 10^−5^, p = 0.071), MV_2_ (coefficient = 1.2 × 10^−3^, p = 0.057), MV_3_ (coefficient = 2.1 × 10^−3^, p = 0.089), LogMAR (coefficient = −9.4 × 10^−4^, p = 0.18), MS_0_ (coefficient = 0.011, p = 0.47), MS_2_ (coefficient = −0.0028, p = 0.86), MS_4_ (coefficient = −0.0045, p = 0.79) and MS_6_ (coefficient = −7.7 × 10^−3^, p = 0.60), except for SRDH (coefficient = 0.51, p = 0.048, linear regression).

## Discussion

In the current study, MP-3 VF testing and SD-OCT were measured in patients with CSC. As expected, MP-3-measured VF sensitivity was significantly related to LogMAR VA. LogMAR VA was not significantly related with any structural measurements of CSC, namely the height of SRD, CRT, and MV. On the other hand, a significant relationship was observed between MP-3 measured VF sensitivity and SRDH.

Many previous reports have suggested a significant relationship between LogMAR VA and macular retinal sensitivity. Flaxel *et al*. identified a significant relationship between LogMAR VA and foveal sensitivity, obtained with HFA, in eyes with glaucoma and several macular diseases^32^. Similar results have also been reported between LogMAR VA and microperimetry-measured retinal sensitivity around the macula^33–35^. In agreement with these previous studies, we observed that LogMAR VA was significantly related to MS_0_ and MS_2_. However this significant relationship was not observed when retinal sensitivity is obtained from wider retinal areas (MS_4_ and MS_6_), suggesting LogMAR VA is related to retinal sensitivity near fovea.

The relationship between LogMAR VA and retinal structure is controversial^36–39^. In the current study, no significant relationship was observed between LogMAR VA and SRDH or MV (Table 2). Previous studies included eyes with acute or resolved CSC, whereas only eyes with chronic SRD were enrolled in the current study.

Kim *et al*. investigated the relationship between microperimetry (Spectral OCT/SLO, Toronto, Canada)-measured retinal sensitivity within 2 and 4 degrees of the fovea, and OCT measured macular retinal thickness^35^. As a result, retinal sensitivities declined with an increase in retinal thickness. Chung *et al*. measured macular retinal sensitivity using the Spectral OCT/SLO microperimeter, showing a significant relationship to OCT-measured retinal thickness^33^. MP-3 microperimetry offers an auto-tracking system to ensure accurate stimulation of the retina. Indeed, we have recently evaluated the reproducibility of MP-3 in patients with retinitis pigmentosa, concluding that it offered better reproducibility than the HFA^40^. Interestingly, in the current study, macular retinal sensitivity (MS_0_, MS_2_, MS_4_, and MS_6_) was not significantly correlated with MVs (Table 2). This contradictory result may be attributed to a difference in the patients studied; previous research included eyes with resolved CSC, whereas eyes with SRD were studied in the current study. Other studies have investigated the relationship between microperimetry-measured retinal sensitivity and alternative structural measurements in eyes with CSC. Ojima *et al*. suggested that an irregular RPE line and defects in the inner segment/outer segment junction (IS/OS) line were associated with decreased MP-1-measured retinal sensitivity in resolved CSC eyes^24^. Fujita *et al*. reported that eyes with cone outer segment tips had significantly higher MP-1-measured retinal sensitivity than those without cone outer segment tips in chronic CSC eyes following photodynamic therapy (PDT)^25^. Reports have also suggested a relationship between Spectral OCT/SLO-measured retinal sensitivity and other structural measurements, such as fundus autofluorescence^35,41^. A further study is needed to investigate the relationship between these structural measurements and MP-3 measured retinal sensitivity.

To the best of our knowledge, this is the first report to demonstrate a significant relationship between retinal sensitivity and OCT measured SRDH in chronic CSC eyes. In the current study, MP-3 measured MS_2_, MS_4_ and MS_6_ were significantly correlated with SRDH (Table 2). In contrast, there was no significant relationship between retinal sensitivities and macular volume. These results would suggest, in chronic CSC, a detached retina becomes thinner over time and the survived retinal sensitivity is dependent on this thinning, irrespective of MV. In addition, MS_0_ and LogMAR VA were not significantly related to SRDH. Thus, the deterioration of visual function occurs in a relatively wide area of the retina, and merely measuring VA or foveal sensitivity is not sufficient to properly evaluate visual function in chronic CSC. The disease duration was collected only from 20 eyes, and it was not included in these analyses. We carried out these analyses including the disease duration in the 20 eyes, however almost identical results to the current results were obtained (results not shown in the Results).

Recent reports have indicated that an increase in SRF is associated with an increase in choroidal thickness and choroidal volume in eyes with treatment-naïve CSC^42^. Kim *et al*. also showed a reduction of subfoveal choroidal thickness after spontaneous resolution of SRDH in CSC^43^. These previous studies examined eyes with acute CSC and are therefore inconsistent with the current study. In the current study, CCT was not related to BCVA, retinal sensitivity or MV, but significantly related to SRDH.

A limitation of the current study is that retinal sensitivity was evaluated only with MP-3. A future study should be carried out shedding light on the comparison of the usefulness of MP-3 and HFA in chronic CSC patients. In addition, the current study indicated the usefulness of retinal sensitivity to assess CSC, however, a further evaluation of the correlation between retinal sensitivity and vision-related quality of life in CSC patients would be of interest. Furthermore, it has been reported that OCT parameters such as outer retinal thickness or ellipsoid zone which are associated with retinal sensitivity, in CSC^44^, which was not analyzed in the current study. In the current study 8 eyes had laser treatment. We assume that the effect of this treatment is negligible with respect to the structure-function relationship because the treatment was not performed within 3 mm of the fovea. Further, when we included photodynamic therapy as a fixed effect in the linear regression, the results were largely unchanged.

In conclusion, it is useful to assess retinal sensitivity using MP-3 in patients with chronic CSC, as suggested by the significant relationship with subfoveal SRD height. On the contrary, there was no significant relationship between VA and SRDH.
